# Letter Regarding 2014 Journal of Epidemiology Article by Nazrul Islam Mondal and Mahendran Shitan

**DOI:** 10.2188/jea.JE20140264

**Published:** 2015-06-05

**Authors:** Torsten Selck

**Affiliations:** Faculty of Educational and Social Sciences, University of Oldenburg, Oldenburg, Germany

Dear Prof. Inoue,

In the introduction to their 2014 *Journal of Epidemiology* article,^1^ Nazrul Islam Mondal and Mahendran Shitan state that, “[i]ncreases in life expectancy have been attributed to improvements in sanitation,” (p.117) but fail to either reference or test for this assertion. In their analysis of recent data for poor and middle-income countries, they only focus on fertility, schooling, income, physician density, and human immunodeficiency virus prevalence. According to the World Health Organization’s World Health Statistics 2013 (p.107), improved sanitation is a recognized factor associated with life expectancy and should therefore be included in multivariate regression analysis of life expectancy. Correlating cross-sectional data from the United States Central Intelligence Agency’s World Factbook for 2012 yields a coefficient of 69% (*N* = 161) for improved sanitation and life expectancy; by contrast, income per capita, which has been regarded since Samuel Preston’s ground-breaking 1975 study in Population Studies as the driving force in explaining life expectancy in a cross-national setting, only scores 61% (*N* = 165).

Yours sincerely,

Torsten J. Selck
